# Impact of Polydeoxyribonucleotides on the Morphology, Viability, and Osteogenic Differentiation of Gingiva-Derived Stem Cell Spheroids

**DOI:** 10.3390/medicina60101610

**Published:** 2024-10-01

**Authors:** Heera Lee, Somyeong Hwa, Sunga Cho, Ju-Hwan Kim, Hye-Jung Song, Youngkyung Ko, Jun-Beom Park

**Affiliations:** 1 Department of Periodontics, College of Medicine, The Catholic University of Korea, Seoul 06591, Republic of Korea; yysmjj@naver.com (H.L.); somyeong.hwa@gmail.com (S.H.); tjda99@naver.com (S.C.); juhwank33@naver.com (J.-H.K.); ko_y@catholic.ac.kr (Y.K.); 2 Department of Medicine, Graduate School, The Catholic University of Korea, Seoul 06591, Republic of Korea; 3 Dental Implantology, Graduate School of Clinical Dental Science, The Catholic University of Korea, Seoul 06591, Republic of Korea; 4 Graduate School of Clinical Dental Science, The Catholic University of Korea, Seoul 06591, Republic of Korea; hjsong55@catholic.ac.kr

**Keywords:** cell differentiation, cell survival, osteogenesis, polydeoxyribonucleotides, stem cells

## Abstract

*Background and Objectives*: Polydeoxyribonucleotides (PDRN), composed of DNA fragments derived from salmon DNA, is widely recognized for its regenerative properties. It has been extensively used in medical applications, such as dermatology and wound healing, due to its ability to enhance cellular metabolic activity, stimulate angiogenesis, and promote tissue regeneration. In the field of dentistry, PDRN has shown potential in promoting periodontal healing and bone regeneration. This study aims to investigate the effects of PDRN on the morphology, survival, and osteogenic differentiation of gingiva-derived stem cell spheroids, with a focus on its potential applications in tissue engineering and regenerative dentistry. *Materials and Methods*: Gingiva-derived mesenchymal stem cells were cultured and formed into spheroids using microwells. The cells were treated with varying concentrations of PDRN (0, 25, 50, 75, and 100 μg/mL) and cultivated in osteogenic media. Cell morphology was observed over seven days using an inverted microscope, and viability was assessed with Live/Dead Kit assays and Cell Counting Kit-8. Osteogenic differentiation was evaluated by measuring alkaline phosphatase activity and calcium deposition. The expression levels of osteogenic markers RUNX2 and COL1A1 were quantified using real-time polymerase chain reaction. RNA sequencing was performed to assess the gene expression profiles related to osteogenesis. *Results*: The results demonstrated that PDRN treatment had no significant effect on spheroid diameter or cellular viability during the observation period. However, a PDRN concentration of 75 μg/mL significantly enhanced calcium deposition by Day 14, suggesting increased mineralization. RUNX2 and COL1A1 mRNA expression levels varied with PDRN concentration, with the highest RUNX2 expression observed at 25 μg/mL and the highest COL1A1 expression at 75 μg/mL. RNA sequencing further confirmed the upregulation of genes involved in osteogenic differentiation, with enhanced expression of RUNX2 and COL1A1 in PDRN-treated gingiva-derived stem cell spheroids. *Conclusions*: In summary, PDRN did not significantly affect the viability or morphology of gingiva-derived stem cell spheroids but influenced their osteogenic differentiation and mineralization in a concentration-dependent manner. These findings suggest that PDRN may play a role in promoting osteogenic processes in tissue engineering and regenerative dentistry applications, with specific effects observed at different concentrations.

## 1. Introduction

Polydeoxyribonucleotides (PDRN) consists of DNA fragments with molecular weights ranging from 50 to 1500 kDa, derived from salmon DNA, and has gained significant attention in medical applications, including skincare and esthetic treatments [1]. PDRN is widely recognized for its role in skin rejuvenation and is commonly used in anti-aging products due to its ability to improve skin elasticity and reduce the appearance of wrinkles [2]. It was reported to stimulate the metabolic activity of skin cells, promoting enhanced skin health and appearance [3]. Additionally, PDRN exhibits wound healing and anti-inflammatory properties by activating the adenosine A2A receptor and salvage pathways [4]. It also stimulates the production of vascular endothelial growth factor (VEGF), particularly in conditions of impaired tissue perfusion [5]. Due to its regenerative capabilities, PDRN is widely used in treatments aimed at tissue repair and wound healing [6], including the treatment of diabetic ulcers and other chronic, slow-healing wounds [7,8]. PDRN is frequently incorporated into serums, creams, and other skincare formulations [9].

In the dental field, PDRN has been applied for its regenerative and healing properties, particularly in the treatment of periodontal diseases, where it supports the healing of periodontal tissues [10]. Studies have shown that PDRN combined with demineralized dentin matrix can induce new bone formation, as well as the generation of osteoblasts and fibroblasts in soft tissues, as demonstrated in a nude mouse model [11]. Furthermore, PDRN has been shown to improve glucocorticoid-induced osteoporosis in a rat model by enhancing the expression of A2A receptors and vascular endothelial growth factor [12]. It was suggested as a potential therapeutic option for managing medication-related osteonecrosis of the jaw due to its ability to reduce inflammation and increase vascularization [13]. PDRN-treated samples exhibited lower necrotic bone percentages and a higher number of blood vessels, along with increased osteoclast production. Local administration of high-dose PDRN (8 mg/kg) significantly improved osteonecrosis resolution [14]. PDRN has also been explored for its use in treating oral mucositis and other mucosal injuries [15]. In one study, PDRN combined with a xenogeneic collagen matrix for gingival phenotype modification yielded results comparable to autogenous connective tissue grafts, suggesting that PDRN-soaked xenogeneic collagen matrix could provide similar benefits for gingival augmentation [16]. This study aims to evaluate the impact of PDRN on the morphology, viability, osteogenic differentiation, and mineralization of human mesenchymal stem cell spheroids. The null hypothesis is that PDRN does not influence cell viability or osteogenic differentiation.

## 2. Materials and Methods

### 2.1. Utilizing Gingiva-Derived Mesenchymal Stem Cells

This study protocol was reviewed and approved by the Institutional Review Board of Seoul St. Mary’s Hospital, College of Medicine, The Catholic University of Korea (approval numbers: KC24SISI0304, 17 May 2023, and KC24SISI0351, 12 July 2024). All experiments were conducted in accordance with the ethical standards outlined in the Declaration of Helsinki. Cell cultures were maintained in an incubator at 37 °C with 95% air and 5% CO_2_, and the culture media were replaced every two to three days.

### 2.2. Stem Cell Spheroid Fabrication

Gingiva-derived mesenchymal stem cells were seeded at a density of 1 × 10^6^ cells/well in 600 µm diameter concave microwells (StemFIT 3D; MicroFIT, Seongnam-si, Gyeonggi-do, Republic of Korea) made of silicon elastomer [17]. The cells were cultured in osteogenic media with PDRN (Genoss, Suwon, Republic of Korea) at final concentrations of 0, 25, 50, 75, and 100 μg/mL. Morphological observations were conducted using an inverted microscope (CKX41SF, Olympus Corporation, Tokyo, Japan) on Days 0, 1, 3, 5, and 7.

### 2.3. Assessment of Qualitative and Quantitative Cell Viability

Qualitative cell viability was evaluated using the Live/Dead Kit assay (Molecular Probes, Eugene, OR, USA) on Days 1 and 7 [18]. Spheroids were viewed at 100× magnification using a fluorescence microscope (Axiovert 200, Carl Zeiss, Oberkochen, Germany) after 60 min of incubation at room temperature. Quantitative viability was assessed with the Cell Counting Kit-8 (Dojindo, Tokyo, Japan) on Days 1, 3, 5, and 7 [19].

### 2.4. Levels of Alkaline Phosphatase Activity and Calcium Deposition 

The evaluation of osteogenic differentiation was carried out by measuring alkaline phosphatase activity and assessing calcium deposition through an anthraquinone dye assay. Cell spheroids cultivated in osteogenic medium were collected on Days 7 and 14 for analysis. Alkaline phosphatase activity was measured with a commercial kit (K412-500, BioVision, Inc., Milpitas, CA, USA), and absorbance at 405 nm was recorded after mixing the cell lysates with the assay reagent (K412-500; BioVision, Inc.). On both Days 7 and 14, calcium deposition was assessed using an anthraquinone dye assay. Stem cell spheroids were washed, fixed, and stained with Alizarin Red S at room temperature for 30 min. After extraction, cetylpyridinium chloride was used to quantify the bound dye.

### 2.5. RNA Extraction and Real-Time Quantitative Polymerase Chain Reaction for RUNX2 and COL1A1

On Day 7, total RNA was extracted using a commercially available kit (Thermo Fisher Scientific, Inc., Waltham, MA, USA), and RNA quantity was measured using a spectrophotometer (ND-2000, Thermo Fisher Scientific, Inc.) and bioanalyzer (Agilent 2100, Agilent Technologies) [20]. Reverse transcription was performed using SuperScript II (Invitrogen, Carlsbad, CA, USA). Primer sequences were designed from GenBank: RUNX2 (accession no. NM_001024630.3), COL1A1 (accession no. NM_000088.3), and β-actin (accession no. X00351.1). Real-time quantitative polymerase chain reaction (qPCR) was conducted to quantify the expression levels of RUNX2 and COL1A1 [21,22].

### 2.6. Isolation, Library Preparation, Sequencing, and Data Analysis

Total RNA was isolated from gingiva-derived stem cells using TRIzol^®^ reagent (Invitrogen; Thermo Fisher Scientific, Inc.). RNA quality was assessed via Agilent 2100 bioanalyzer (Agilent Technologies, Inc.), and RNA quantification was performed with a spectrophotometer (ND-2000; Thermo Fisher Scientific, Inc.). Libraries were prepared from total RNA using the NEBNext Ultra II Directional RNA-Seq kit (New England BioLabs, Inc., Ipswich, MA, USA). The isolated mRNAs were reverse-transcribed into cDNA, according to the manufacturer’s instructions (New England BioLabs, Inc.). Quantification was performed using the library quantification kit, and library concentration was measured using Tape Station HS D1000 Screen Tape (Agilent Technologies, Inc., Santa Clara, CA, USA). The library loading volume and loading concentration were 150 µL and 300 pM, respectively. Sequencing was performed using Illumina Novaseq 6000 (Illumina, Inc., San Diego, CA, USA). The NovaSeq 6000 S4 Reagent kit v1.5 (300 cycles; Illumina, Inc.) was used as the sequencing reagent kit. Running format was set at PE100bP. Quality control of raw sequencing data was performed, and low-quality reads (<Q20) were removed [23]. Estimation of gene expression levels and normalization of the values were performed [24]. Pathway analysis was performed on differentially expressed genes using the Kyoto Encyclopedia of Genes and Genomes mapping tool [25]. Data mining and graphic visualization were performed afterwards.

### 2.7. Statistical Analysis

Data are presented as mean ± standard deviation. Normality and equality of variances were verified before performing comparisons between groups using one-way ANOVA followed by Tukey’s post hoc test (SPSS 12 for Windows, SPSS Inc., Chicago, IL, USA). All experiments were conducted in triplicate.

## 3. Results

### 3.1. Morphological Analysis of Human Gingiva-Derived Mesenchymal Stem Cell Spheroids

**Figure 1:**
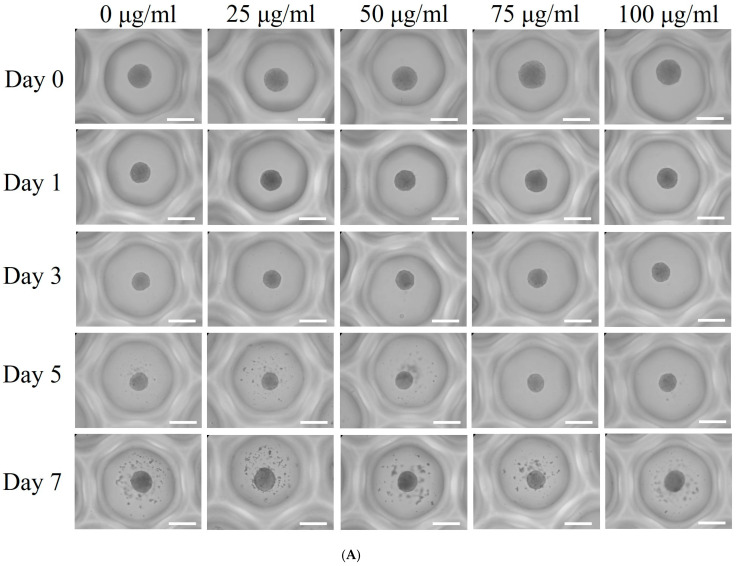

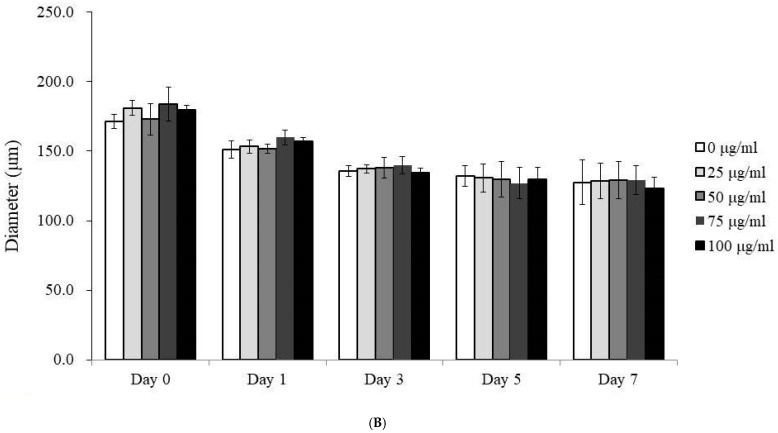
Morphological evaluation of stem cell spheroids. (**A**) Representative images of spheroids treated with PDRN at concentrations of 0, 25, 50, 75, and 100 μg/mL on Days 0, 1, 3, 5, and 7. Scale bar = 200 μm (original magnification ×200). (**B**) Changes in spheroid diameter over time, measured on Days 0, 1, 3, 5, and 7.

Figure 1A presents a time-course and dose–response study of gingiva-derived stem cells’ morphology in an osteogenic medium when subjected to varying concentrations of PDRN. The images depict a clear progression in morphological characteristics with increasing concentration and time. On Day 0, spheroids across all concentrations maintained a relatively uniform and spherical shape. By Day 1, similar trends were seen with no significant concentration-dependent response. By Days 3 and 5, the spheroids had similar shapes and a consistently rounded shape but were smaller in size.

In terms of diameter, all spheroids exhibited similar dimensions across all treatment groups on Day 0 (Figure 1B). By Day 1, a notable reduction in median diameter was observed, which became more pronounced by Day 5 (*p* < 0.05). However, PDRN treatment across different concentrations did not significantly influence spheroid diameter over the five-day period (*p* > 0.05).

### 3.2. Qualitative and Quantitative Assessment of Cell Viability

**Figure 2:**
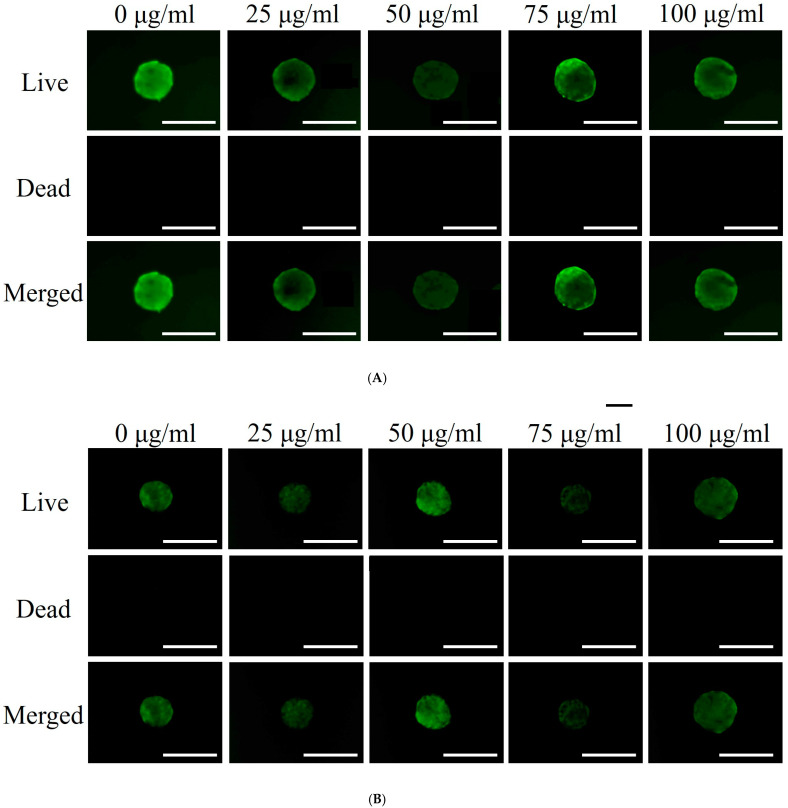

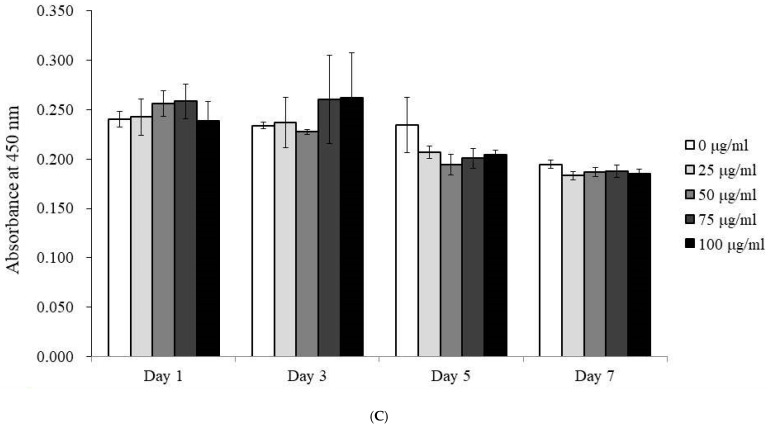
Cellular viability assessment. (**A**) Optical, live, dead, and merged images of stem cell spheroids on Day 1. Scale bar = 200 μm (original magnification ×200). (**B**) Optical, live, dead, and merged images of stem cell spheroids on Day 7. Scale bar = 200 μm (original magnification ×200). (**C**) Quantitative analysis of cell viability using the Cell Counting Kit-8 on Days 1, 3, 5, and 7. Varying concentrations of PDRN did not have a significant effect on cell viability over the seven-day period (*p* > 0.05).

Qualitative cell viability was assessed using the Live/Dead Kit assay on Days 1 and 7, as shown in Figure 2A. Fluorescence images revealed no significant differences in cell viability across the various PDRN concentrations on Day 1. Figure 2B shows similar viability trends on Day 7. Quantitative cell viability was measured using the Cell Counting Kit-8 on Days 1, 3, 5, and 7 (Figure 2C). On Day 1, median viability across all groups was similar, as indicated by comparable absorbance values. A slight variation was observed on Day 3, followed by a decrease in absorbance on Day 5, suggesting reduced cell viability over time. By Day 7, a more pronounced decrease was evident. However, different PDRN concentrations did not significantly impact cell viability over the seven days (*p* > 0.05).

### 3.3. Alkaline Phosphatase Activity and Calcium Deposition

**Figure 3:**
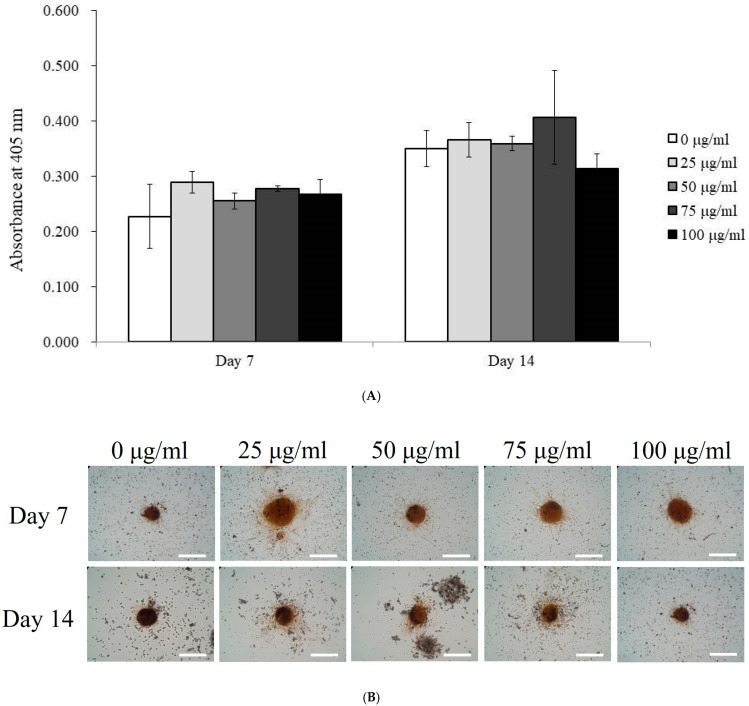

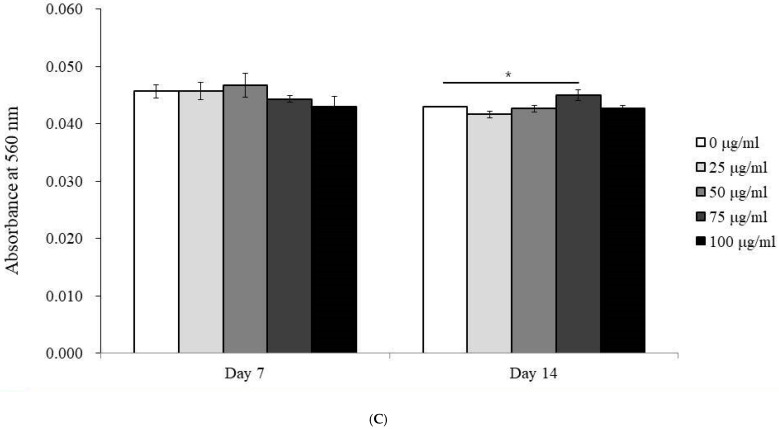
Osteogenic differentiation in PDRN-treated spheroids. (**A**) Alkaline phosphatase activity in PDRN-treated spheroids measured on Days 7 and 14. (**B**) Evaluation of calcium deposition in PDRN-treated spheroids on Days 7 and 14. (**C**) Quantitative analysis of calcium deposition in spheroids treated with different PDRN concentrations. * *p* < 0.05 on day 14 compared to the time-matched unloaded group.

Alkaline phosphatase (ALP) activity, an early marker of osteogenesis, was measured in spheroids treated with PDRN at concentrations of 0, 25, 50, 75, and 100 μg/mL on Days 7 and 14 (Figure 3A). On Day 7, ALP activity was relatively uniform across all concentrations, with no significant differences observed.

Calcium deposition, a marker of late-stage osteogenesis, was assessed on Days 7 and 14 (Figure 3B,C). On Day 7, there were no significant differences in calcium deposition between treatment groups. However, by Day 14, the 75 μg/mL PDRN-treated group showed a significant increase in calcium deposition (*p* < 0.05), indicating enhanced mineralization in this group.

### 3.4. Expression of RUNX2 and COL1A1 mRNA by qPCR

**Figure 4:**
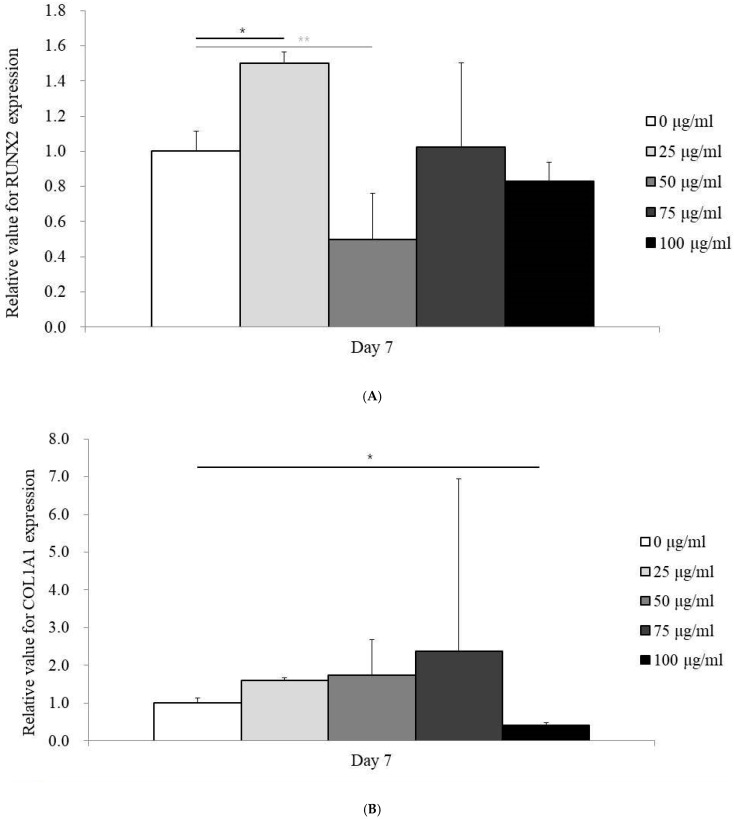
mRNA expression analysis. (**A**) Quantification of RUNX2 mRNA expression using qPCR on Day 7. * *p* < 0.05 on day 7 compared to the unloaded group. ** *p* < 0.05 compared to the unloaded group. (**B**) Quantification of COL1A1 mRNA expression using qPCR on Day 7. * *p* < 0.05 compared to the unloaded group.

RUNX2 and COL1A1 mRNA expression levels were evaluated by qPCR on Day 7 following treatment with PDRN at varying concentrations (0, 25, 50, 75, and 100 µg/mL). The expression of RUNX2 was highest in the 25 µg/mL group, with levels of 1.499 ± 0.066, compared to 1.000 ± 0.116 in the control group (*p* < 0.05) (Figure 4A).

Similarly, COL1A1 expression peaked at 75 µg/mL (2.369 ± 4.579), with the control group showing an expression level of 1.000 ± 0.138 (*p* < 0.05) (Figure 4B). These results suggest that specific PDRN concentrations enhanced the expression of osteogenic markers.

### 3.5. RNA Sequencing Data Analysis

**Figure 5:**
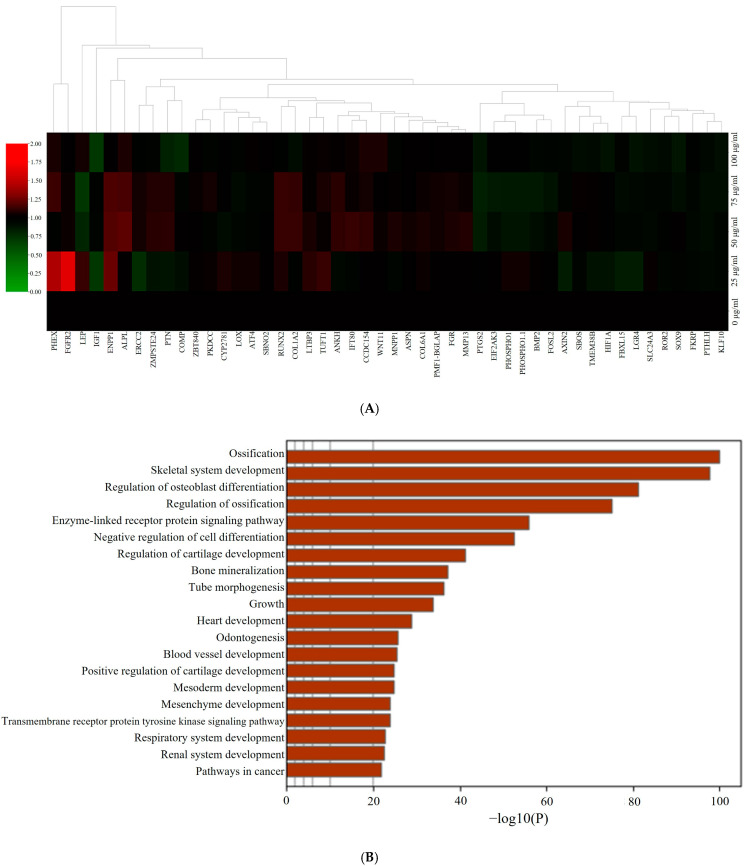

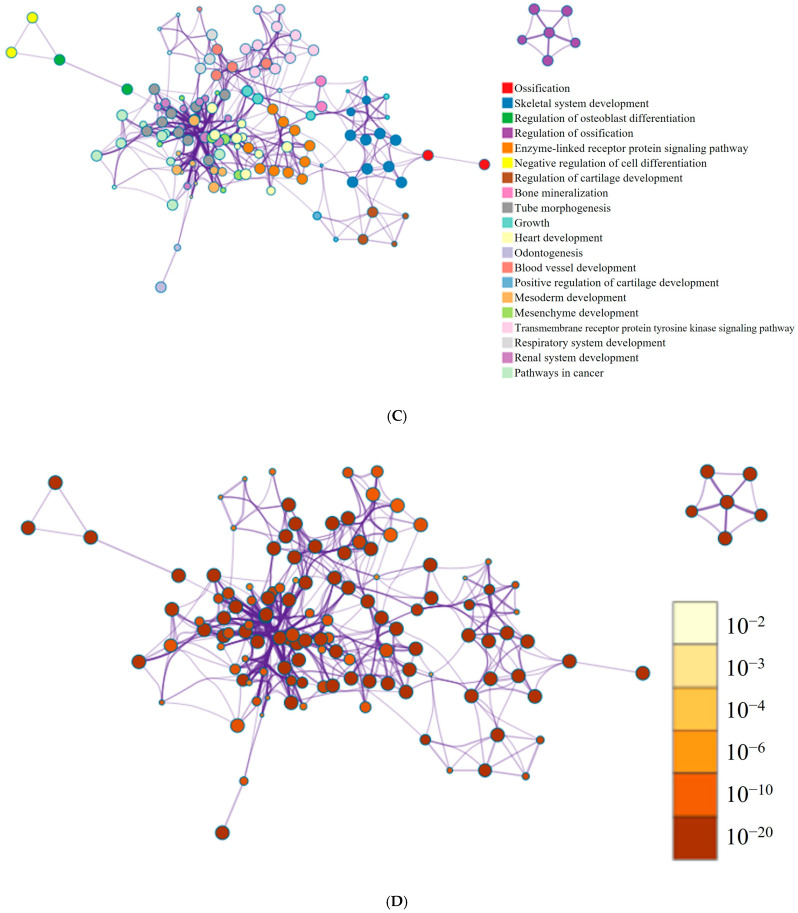

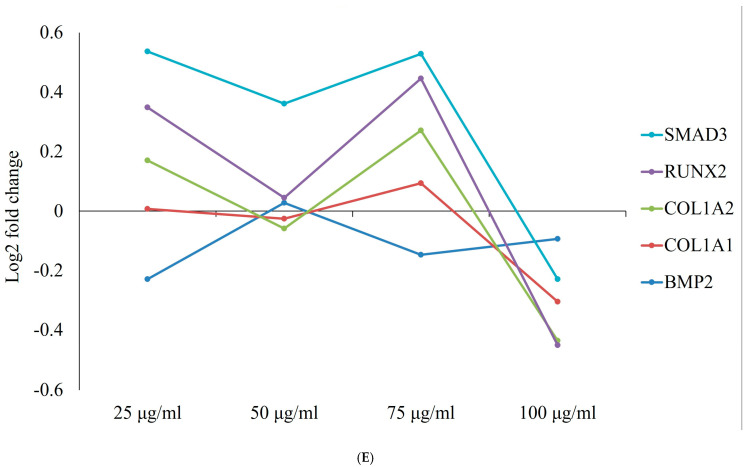
RNA sequencing analysis. (**A**) Cluster heatmap with hierarchical clustering showing osteogenic differentiation in PDRN-treated gingiva-derived stem cell spheroids. (**B**) Functional enrichment analysis. (**C**) Pathway relationship network colored by cluster identity. (**D**) Pathway relationship network colored by p-value significance. (**E**) Gene expression profiling for regulation of osteogenic differentiation in PDRN-treated stem cell spheroids.

Figure 5A presents a hierarchical clustering heatmap illustrating gene expression profiles related to osteogenesis in the PDRN-treated spheroids. Red indicates high expression, while green denotes low expression. Dendrograms at the top represent gene clustering based on expression similarity. Further pathway analysis, shown in Figure 5B, suggested that PDRN influenced key osteogenic pathways. Enriched terms were filtered by hypergeometric *p*-values and enrichment factors, and clustered into a hierarchical tree with a kappa score threshold of 0.3.

Figure 5C depicts the relationships between enriched pathways as a network, where node size correlates with the number of input genes, and edge thickness reflects similarity scores. The same network is shown in Figure 5D, with nodes colored by p-value, indicating the statistical significance of the terms. Figure 5E shows gene expression profiling for osteogenic differentiation, highlighting the dose-dependent effects of PDRN on osteogenic gene expression. RUNX2 and COL1A1 expression peaked at 75 µg/mL, supporting the observed increase in osteogenesis at this concentration.

## 4. Discussion

This study investigated the effects of polydeoxyribonucleotides (PDRN) on the osteogenic differentiation and mineralization of human mesenchymal stem cell spheroids.

In terms of spheroid morphology, a reduction in spheroid size was observed over time, which was consistent across all PDRN concentrations. This indicates that PDRN does not exert a concentration-dependent effect on spheroid morphology, as the spheroids maintained a stable, rounded shape throughout the experimental period. Similarly, the decline in cell viability over time, expected in long-term cultures, was not significantly influenced by PDRN concentration, suggesting that PDRN does not impair cell viability or induce cytotoxicity at any dose.

In relation to osteogenic differentiation, a significant increase in calcium deposition was noted at a PDRN concentration of 75 μg/mL by Day 14. This enhanced mineralization is a critical indicator of osteogenesis, suggesting that PDRN may promote the later stages of bone formation. While a clear dose-dependent effect on osteogenic marker expression was not evident, the mRNA levels of RUNX2 and COL1A1 were elevated at specific concentrations, with RUNX2 peaking at 25 μg/mL and COL1A1 at 75 μg/mL. RUNX2 plays a pivotal role in the early stages of osteogenic differentiation by directing mesenchymal stem cells towards the osteogenic lineage, while COL1A1 encodes type I collagen, essential for the extracellular matrix and bone tissue development. The observed upregulation of these markers suggests that PDRN may modulate different phases of osteogenic differentiation, promoting both lineage commitment and matrix formation essential for bone maturation.

RNA sequencing further revealed that PDRN treatment may influence gene expression profiles associated with osteogenesis [26,27], with the clustering of differentially expressed genes related to bone formation suggesting that PDRN could regulate molecular pathways involved in osteogenic differentiation. These findings imply that PDRN’s effects may extend beyond individual osteogenic markers to broader gene networks, though further research is necessary to fully elucidate its role in bone regeneration.

PDRN is well documented for its ability to stimulate cell growth and tissue regeneration [28], and its benefits in wound healing and reducing inflammation have been widely acknowledged [29]. PDRN promotes angiogenesis, a vital process for supplying oxygen and nutrients to regenerating tissues, including bone, and activates the adenosine A2A receptor, which plays a key role in anti-inflammatory processes and tissue regeneration [30]. The activation of adenosine A2A receptors by PDRN promotes sustained angiogenesis and anti-inflammatory effects, both of which are essential for maintaining blood supply and reducing inflammation throughout the prolonged phases of tissue regeneration. This may allow for a more sustained healing process compared to the shorter-term effects of platelet-rich plasma and platelet-rich fibrin, which release growth factors quickly but have a less prolonged impact [31]. Since our results suggest that PDRN may enhance osteogenic differentiation, they underscore its potential for contributing significantly to the long-term regeneration of both bone and soft tissues. Moreover, PDRN has been shown to enhance the expression of various growth factors, such as VEGF, making it a valuable adjunct in procedures requiring tissue regeneration, including those in dentistry [32].

PDRN was applied at varying concentrations based on the experimental design. In one study, PDRN at a concentration of 100 μg/mL led to a 21% increase in osteoblast growth after six days compared to the control group [33]. Bone marrow mesenchymal stem cells cultured with 100 μg/mL of PDRN showed upregulated gene expression related to osteogenesis and angiogenesis, while inflammatory markers were reduced [34]. In a previous animal study, demineralized dentin matrix combined with 10 μg/mL PDRN was implanted subcutaneously into the dorsal region of nude mice. This combination successfully induced bone regeneration in the model [11]. Another study using a beagle dog model for lateral sinus floor elevation involved the application of a collagenated synthetic bone graft combined with PDRN at a concentration of 1.875 mg/mL, which resulted in enhanced early new bone formation and greater bone-to-implant contact [28]. Further research investigated the use of xenogeneic collagen matrix with PDRN for gingival phenotype modification in a mongrel dog model, comparing it to autogenous connective tissue grafts. A coronally positioned flap was performed using either a subepithelial connective tissue graft or collagen matrix with 2.0 mg/mL PDRN [16]. 

In this study, the relative mRNA expression levels of RUNX2 and COL1A1 were analyzed in stem cell spheroids cultured in osteogenic media and exposed to varying concentrations of PDRN. RUNX2 is a crucial transcription factor that plays a pivotal role in osteogenesis by regulating the differentiation of osteoblasts, which are essential for bone formation [35]. Assessing RUNX2 expression provides a comprehensive and direct evaluation of osteogenic activity, as it functions as both a master regulator and mediator of multiple signaling pathways involved in bone development [36]. On the other hand, COL1A1 serves as an early marker of osteoblast differentiation. It is one of the first genes expressed when progenitor cells commit to the osteogenic lineage, making it a key indicator of the initial stages of bone formation [37]. Additionally, type I collagen, encoded by COL1A1, is critical for bone tissue formation [38]. It provides the structural scaffold for mineral deposition, which is essential for maintaining bone strength and rigidity. By monitoring changes in COL1A1 expression, researchers can gain valuable insights into the effectiveness of osteogenic treatments, particularly during the early phases of bone formation.

In a combinatory approach, magnesium hydroxide and bone extracellular matrix were incorporated into a poly(lactic-co-glycolic) acid scaffold, immobilized with a nanocomplex containing 1 mg/mL PDRN and 1 mg/mL bone morphogenetic protein-2, yielding promising results of osteogenesis, angiogenesis, and anti-inflammatory activity [39]. Additionally, varying concentrations of PDRN (0.1, 1, 5, and 10 mg/mL) and recombinant human bone morphogenetic protein-2 (0.01, 0.05, and 0.1 mg/mL) were evaluated for their bone regeneration capacity and mechanical properties using a white rabbit calvarial defect mode [40]. 

Recent studies have explored the potential of PDRN in different medical applications. For instance, PDRN has been shown to stimulate osteoblast growth and increase gene expression related to osteogenesis and angiogenesis while decreasing inflammatory markers in bone marrow mesenchymal stem cells. Additionally, PDRN has been successfully used in combination with demineralized dentin matrix, leading to new bone formation in animal models, and in dental procedures such as sinus floor elevation, where it enhanced early bone formation and increased bone-to-implant contact [11]. Furthermore, innovative delivery methods, such as encapsulating PDRN in chitosan polyplexes, have been proposed to improve its therapeutic potential, particularly in wound healing applications [41].

There are several limitations to this study. First, the investigation was conducted in a controlled laboratory setting using concave microwells, which may not fully capture the complexity of the in vivo environment [40]. This could affect the direct translation of the findings into practical applications [42]. Additionally, while the study focuses on the short-term effects of PDRN on viability and differentiation, it does not address the long-term impact of PDRN exposure on stem cell spheroids [3,43], which is crucial for clinical applications. Although a range of PDRN concentrations was evaluated, the biological significance of each concentration may not have been fully explored [6]. These limitations highlight the need for further research, particularly using more physiologically relevant models and longer study durations, to validate these findings and ensure the safety and efficacy of PDRN in regenerative medicine applications.

The ability of PDRN to enhance osteogenesis, angiogenesis, and tissue regeneration has been demonstrated in various preclinical studies, with promising results observed in both bone and soft tissue repair [16,28]. This study demonstrated that PDRN may play a valuable role in promoting the differentiation of stem cell spheroids, particularly in the context of osteogenic differentiation and mineralization. However, clinical trial data, particularly in the dental field, remain limited, making it difficult to establish standardized guidelines for its use. More comprehensive and diverse clinical trials are needed to better understand PDRN’s efficacy across a broader range of treatments. Additionally, future research could explore the combination of PDRN with other regenerative treatments, such as stem cell therapy or biologically active scaffolds, to optimize healing and tissue regeneration outcomes.

## 5. Conclusions

In conclusion, the results of this study suggest that PDRN may play a valuable role in promoting the differentiation of stem cell spheroids, particularly in the context of osteogenic differentiation and mineralization. Further research is warranted to fully understand its potential applications in tissue engineering and regenerative medicine.

## Data Availability

Original contributions presented in the study are included in the article; further inquiries can be directed to the corresponding author.
